# Robust Plasma Cell Response to Skin-Inoculated Dengue Virus in Mice

**DOI:** 10.1155/2021/5511841

**Published:** 2021-04-26

**Authors:** Raúl A. Maqueda-Alfaro, Edith Marcial-Juárez, Juana Calderón-Amador, Julio García-Cordero, Mariana Orozco-Uribe, Felipe Hernández-Cázares, Uziel Medina-Pérez, Luvia E. Sánchez-Torres, Adriana Flores-Langarica, Leticia Cedillo-Barrón, Juan C. Yam-Puc, Leopoldo Flores-Romo

**Affiliations:** ^1^Department of Cell Biology, Center for Research and Advanced Studies, The National Polytechnic Institute, Cinvestav-IPN, Av. IPN 2508, San Pedro Zacatenco, Gustavo A. Madero, 07360 Mexico City, Mexico; ^2^Department of Molecular Biomedicine, Center for Research and Advanced Studies, The National Polytechnic Institute, Cinvestav-IPN, Av. IPN 2508, San Pedro Zacatenco, Gustavo A. Madero, 07360 Mexico City, Mexico; ^3^Universidad de la Cañada, Oaxaca State Universities (SUNEO), Carretera Teotitlán-San Antonio Nanahuatipán Km 1.7, 68540 Teotitlán de Flores Magón, Oaxaca, Mexico; ^4^Departamento de Inmunología, Escuela Nacional de Ciencias Biológicas, Instituto Politécnico Nacional, Prolongación de Carpio y Plan de Ayala s/n, Ciudad de México 11400, Mexico; ^5^Institute of Immunology and Immunotherapy, College of Medical and Dental Sciences, University of Birmingham, Vincent Dr, Edgbaston B15 2TT, Birmingham, UK

## Abstract

Dengue is a worldwide expanding threat caused by dengue virus (DENV) infection. To date, no specific treatment or effective vaccine is available. Antibodies produced by plasma cells (PCs) might be involved concomitantly in protection and severe dengue immunopathology. Although a massive appearance of PCs has been reported during acute DENV infection in humans, this response has been poorly characterized. Here, we show the dynamic of PC generation in immune-competent mice cutaneously inoculated with DENV compared with two control experimental groups: mice inoculated with inactivated DENV or with PBS. We found that PC numbers increased significantly in the skin-draining lymph node (DLN), peaking at day 10 and abruptly decreasing by day 14 after DENV inoculation. Class-switched IgG^+^ PCs appeared from day 7 and dominated the response, while in contrast, the frequency of IgM^+^ PCs decreased from day 7 onwards. Even though the kinetic of the response was similar between DENV- and iDENV-inoculated mice, the intensity of the response was significantly different. Interestingly, we demonstrated a similar PC response to virus antigens (E and prM) by ELISPOT. *In situ* characterization showed that PCs were distributed in the medullary cords and in close proximity to germinal centers (GCs), suggesting both an extrafollicular and a GC origin. Proliferating PCs (Ki-67^+^) were found as early as 3-day postinoculation, and in-depth analysis showed that these PCs were in active phases of cell cycle during the kinetic. Finally, we found a progressive appearance of high-affinity neutralizing DENV-specific IgG further supporting GC involvement. Of note, these antibodies seem to be highly cross-reactive, as a large proportion recognizes Zika virus (ZIKV). The strong PC response to skin-inoculated DENV in this work resembles the findings already described in humans. We consider that this study contributes to the understanding of the *in vivo* biology of the humoral immune response to DENV in an immunocompetent murine model.

## 1. Introduction

Dengue virus (DENV) is an important viral pathogen affecting 390 million people worldwide yearly [1]. Currently, DENV is endemic in over 100 tropical and subtropical countries, resulting in 40% of the global population at risk of infection. DENV has 4 serotypes (DENV1-4) that are transmitted by female *Aedes aegypti* and *albopictus* mosquitoes. In most cases, dengue disease is asymptomatic or manifests in a range of febrile illnesses, from mild to classical dengue fever (DF) with severe headache and joint pain. However, the infection can evolve to severe dengue (SD) forms, dengue hemorrhagic fever/dengue shock syndrome (DHF/DSS), characterized by thrombocytopenia, vascular leakage, and hemorrhage, leading to organ failure and death [2].

It has been suggested that one of the main risk factors for SD is the secondary infection with a heterologous DENV serotype [3–6]. While different factors like age, time period between infections, host genetic background, and virus serotype and isolates contribute to the disease outcome, the level and characteristics of preexisting anti-DENV antibodies have been associated with the severity of the disease [3, 7–9]. One of the leading hypotheses for this is the antibody-dependent enhancement (ADE) of DENV infection, where cross-reactive sub- or nonneutralizing antibodies facilitate the entry, and consequently the replication, of the virus in Fc*γ* receptor- (Fc*γ*R-) bearing cells [9–11].

Antibodies generated during a natural primary DENV infection provide a long-term protection to the same serotype but only offer short-term protection to other serotypes [12–14]. Besides the important role of antibodies not only in host protection but also in dengue pathogenesis, the dynamics of antibody-secreting plasma cell (PC) generation during DENV infection are poorly characterized [15]. Few and recent studies have described a massive and rapid appearance of antibody-secreting plasmablasts (PBs), accounting for up to 80% of the circulating B cells in the blood of human patients during the acute phase of DENV infection [15–18]. The majority of these PBs are DENV cross-reactive, and this response seems to be independent of the severity of the disease (DF or SD) [17]. Evidence also suggests that some of those PBs produced during the acute response to DENV are part of a polyclonal response of polyreactive natural IgG B cells. Additionally, PBs generated during ongoing secondary dengue infections are also highly cross-reactive and derived from memory B cells (MBCs) [19, 20].

Activated B cells can undergo a rapid extrafollicular PC differentiation or affinity maturation in germinal center (GC) reactions on secondary lymphoid organs (SLO) [21, 22]. In GCs, IgG class-switched B cell clones are selected based on their BCR affinity to the immunizing antigen. Selected clones survive and differentiate into either long-lived PCs (LLPCs) or MBCs. LLCPs home to survival niches like the bone marrow, where they secrete high-affinity antibodies [23, 24]. On the other hand, antigen-activated B cells can proliferate and then differentiate, outside of the follicle (extrafollicular response), mainly into nonswitched IgM-secreting PCs [21]. The generation of extrafollicular and follicular PCs during ongoing infections leads to immediate and long-term protection; meanwhile, MBC activation provides rapid responses against reexposure to the same antigen [21–24].

Although PCs seem to dominate the B cell response during acute DENV infection and their function is intimately related to host protection or infection enhancement, the mechanisms leading to the origin of these cells remain unclear. Given the understandable difficulties to study the basic cellular mechanisms of PC generation during dengue infection in humans, the use of suitable *in vivo* animal models is needed. Immune-deficient and humanized mice have been extensively used in the study of dengue in order to sustain viral replication and clinical manifestations [25, 26] but may not be the most appropriate model for the analysis of immune responses. We have used immune-competent mice, where the dose and route of infection mimic what occurs in natural infections [27–29]. This model allows us to analyze the immune response in an unperturbed environment.

We have previously shown that DENV induces a large GC response with antigen-specific GC B cells for either precursor membrane (prM) or envelope (E) proteins in immune-competent mice following an intradermal, relatively low dose of DENV inoculation [27]. However, the dynamics of PCs to DENV in this model remain unknown. In this work, we have evaluated the kinetics of PC generation through their expression of CD138 and Ly6C, the proportion of class-switched cells, their transit through the different stages of the cell cycle, apoptosis rate, and their specificity to DENV and E or prM structural proteins. Our results resemble the observations described in human patients with dengue. We consider that this animal model can be importantly used for a better understanding of the *in vivo* biology of the humoral immune response to DENV.

## 2. Materials and Methods

### 2.1. Mice and Immunizations

Specific pathogen-free BALB/c adult (6-8 weeks) male mice were provided by the animal facilities (UPEAL) at the Center for Research and Advances Studies of the National Polytechnic Institute (CINVESTAV-IPN). As stablished previously [27, 29], mice were intradermally (i.d.) inoculated in the inguinal region with one dose of 6 × 10^4^ plaque-forming units (PFU) of active DENV serotype 2 New Guinea reference strain and a boost (6 × 10^4^ PFU) at day 7. Thus, mice 3 and 7 d p.i. only received one dose. As controls, we used mice i.d. inoculated with either UV-inactivated DENV (iDENV) or endotoxin-free phosphate buffer solution (PBS). Regional (inguinal) draining lymph nodes (DLNs) were dissected at days 3, 7, 10, 14, 21, and 28 postinoculation (p.i.). In some experiments, mice were i.d. inoculated with alum precipitated DNP-KLH (20 *μ*g). Experiments were performed according to the institutional animal use guidelines for animal care and experimentation (Protocol number: 0070-13, UPEAL-CINVESTAV-IPN).

### 2.2. Preparation of DENV2 and Zika Virus (ZIKV) Stock

DENV2 and ZIKV stock was obtained *in vitro* by infecting mosquito C6/36 or African green monkey kidney-derived Vero cell lines, respectively, with brain extracts from infected neonate mice. Vero or C6/36 cells were grown in Eagle's Minimum Essential Medium (EMEM) supplemented with 10% fetal bovine serum (Gibco, NY, USA), penicillin, amphotericin B, streptomycin, pyruvate, vitamins, and L-glutamine, at 34°C in a 75 cm^2^ culture flask (Corning, NY, USA). Cells were infected at 95% of confluence. Culture supernatant containing DENV or ZIKV was collected 48 h after infection and concentrated with Amicon Centrifugal Filter Units (Merck Millipore, MA, USA). Quantification of infectious virions was performed using a plaque-forming assay in Vero cell line and reported as plaque-forming units (PFU)/mL.

### 2.3. *In Situ* Immunofluorescence Microscopy

DLNs were obtained at different times postinoculation and frozen in tissue freezing medium (Leica, IL, USA) with liquid nitrogen and stored at -70°C until use. 5 *μ*m cryosections were mounted on poly-L-lysine charged slides and fixed in cold acetone for 20 min and stored at -20°C after air-drying. Slides were rehydrated in wash buffer (PBS-0.5% saponin), blocked with 1% BSA in PBS, and incubated 1 hour with primary or fluorochrome-conjugated anti-mouse antibodies (detailed in supplemental Table 1). After two washing steps, secondary antibodies were incubated 1 h at room temperature. Relevant controls were run alongside. Slides were washed 3 times with wash buffer and mounted in antifade mounting medium (ProLong Gold; Invitrogen). Images were acquired with a Zeiss Axio Scan.Z1 slide scanner (Zeiss, Oberkochen, Germany) using a 20x objective and analyzed with Zen 3.0 Blue edition software (Carl Zeiss Microscopy, Jena, Germany).

### 2.4. Flow Cytometry and Cell Cycle Analysis

DLN single-cell suspensions were generated by mechanical disruption and passed through a 70 *μ*m cell strainer. Except for cell cycle staining, cell suspensions were labelled with the Fixable Viability Dye eFluor 450 (eBioscience, Carlsbad, CA) to exclude dead cells from the analysis. Cell suspensions were blocked with universal blocking reagent Power Block (BioGenex, CA, USA) and incubated with the relevant anti-mouse antibodies (detailed in supplemental Table 1). After two washes, cell suspensions were treated for intracellular staining with BD Cytofix/Cytoperm (San Jose, CA) according to manufacturer specifications and further incubated with anti-IgM FITC and anti-IgG APC or anti-active-Caspase3 FITC antibodies. For cell cycle analysis, after surface staining, cell suspensions were treated with the Foxp3/Transcription Factor Fixation/Permeabilization Foxp3 kit (eBioscience, Carlsbad, CA) for intracellular/intranuclear detection of Ki-67. Cells were washed once and then incubated 10 min with Hoechst 33258 (Polysciences, Warrington, PA) for DNA labelling.

Finally, cells were fixed with 1% paraformaldehyde (Sigma-Aldrich, St. Louis, MO) and acquired in a CytoFLEX LX flow cytometer using CytExpert software (Beckman Coulter, Indianapolis, IN). Data was analyzed using FlowJo software vX.0.7 (Ashland, OR).

### 2.5. ELISPOT Analysis

Plates (MultiScreen, Millipore) were coated overnight at 4°C with either 5 × 10^5^ PFU/well of whole DENV2 or 5 *μ*g/mL E or prM recombinant proteins [27] and blocked with 1% BSA for 1 h at 37°C. Single-cell suspensions from DLNs of mice 10 d p.i. were obtained as mentioned above, and 3.5 × 10^5^ cells were added per well in duplicate. Cells were cultured for 5 h at 37°C. After three washes with PBS-0.05% Tween-20 (PBST), the plates were incubated overnight with optimal dilutions of alkaline phosphate-conjugated detection antibodies (anti-IgM or anti-IgG; Southern Biotech). Plates were washed three times, and the reaction was visualized by the addition of Sigma Fast BCIP/NBT (Sigma Aldrich). Spots were counted using the AID ELISPOT Reader System (Autoimmune Diagnostika). Data is presented as IgM or IgG spot-forming units (SFU)/DLN.

### 2.6. Urea Avidity ELISA and Cross-Reactivity Evaluation

Serum was collected by cardiac puncture from terminally anesthetized mice at different time points postinoculation and stored at -20°C until use. 96-well E.I.A./R.I.A. immunoplates (Costar 3590, Cambridge, MA) were coated with 5 × 10^5^ PFU/well of whole DENV2 or 1 *μ*g/mL DNP-KLH diluted in carbonate buffer (pH 9.4) overnight at 4°C in a moist chamber. After three washes and blocking with universal blocking reagent (BioGenex, CA, USA), serial dilutions of serum samples were incubated in duplicate for 2 h at 37°C to evaluate the antibodies both in the absence and in the presence of urea 7 M. After further washing, urea 7 M or PBST was added to each replicate for 10 min at room temperature. Plates were washed two times and incubated 2 h at 37°C with optimal dilution of HRP-conjugated anti-mouse IgG (Vector PI-2000-1). The reaction was visualized by the addition of ABTS substrate (Sigma-Aldrich A1888) for 30 min at 37°C. For cross-reactivity evaluation, immunoplates were coated with 2.5 × 10^5^ PFU of either whole DENV2, ZIKV, or 3 *μ*g/mL SARS-CoV-2 RBD overnight at 4°C in a moist chamber. Procedure continues as mentioned above to evaluate IgG antibodies without the addition of urea 7 M. Absorbance was measured at 405 nm. OD values were plotted against dilution, and smoothed lines were drawn through each dilution series. Relative antibody titers were read as maximal dilution where OD was above an arbitrary threshold. The avidity index was calculated by dividing the relative titer from urea treatment by the relative titer from PBST-only treatment [30, 31].

### 2.7. Viral Neutralization Assay

Sera from DENV2-infected mice and DNP-KLH mice 28 d p.i. were serially diluted in 50 *μ*L RPMI containing 2% (vol/vol) FBS and 1X antibiotics. Then, 2.5 MOI of DENV2 in a volume of 50 *μ*L was added to each serum dilution and incubated for 1 h at 37°C. This serum-virus mix was used to infect 2.5 × 10^4^ Vero cells in 96-well plates in a final volume of 100 *μ*L for 2 h in triplicate. The complexes were then removed and washed with 1X PBS to further replenish with fresh supplemented culture media and incubated for 24 h at 37°C. Next, the cells were treated for intracellular staining with BD Cytofix/Cytoperm (San Jose, CA) for flow cytometric analysis as mentioned above. Cells were stained using the pan-flavivirus 4G2 antibody (anti-Flavivirus E protein, MAB10216, Sigma-Aldrich, Darmstadt, Germany) for 30 min, followed by the anti-mouse IgG Cy3 antibody (A10521; Life Technologies, USA) for 30 min. The percent of infected cells was determined using flow cytometry and was defined as the percentage of 4G2-positive stained cells [32, 33].

### 2.8. Plaque Reduction Neutralization Test

Briefly, 24-well plates were seeded with Vero cells in triplicate and when they reached 80-90% confluence; serial dilutions (1 : 20, 1 : 40 and 1 : 80) were made of sera from DENV2-infected mice and DNP-KLH mice 28 d p.i. in 50 *μ*L RPMI containing 2% (vol/vol) FBS and 1X antibiotics. Then, 50 PFU of DENV2 in a volume of 50 *μ*L was added to each serum dilution and incubated for 1 h at 37°C. This serum-virus complex was used to infect the Vero cells in 24-well plates in a final volume of 100 *μ*L for 2 h in triplicate. Infected Vero cells were then completely overlaid with DMEM containing methylcellulose and maintained for 4 days at 37°C. The overlays were removed with gentle washes and fixed with methanol at 80%, and monolayers were blocked with 5% PBS-milk. After washing, the plates were incubated with mouse IgG 4G2 antibody at 1 : 2000. Subsequently, anti-mouse IgG (H+L) coupled to HRP (G-21040, Invitrogen, Carlsbad, CA, United States) was added to the assay at a 1 : 2000 dilution and incubated for 1 h. Plates were washed, and True Blue™ peroxidase substrate was added to reveal the plaques. Finally, the number of plaques was counted in a stereoscopic microscope.

### 2.9. Statistical Analysis

Values are expressed as mean ± the standard error of the mean (SEM). Statistics were calculated using the GraphPad Prism software ver. 8.0.2 (San Diego, CA) performing one-way or two-way analysis of variance (ANOVA) with the Bonferroni post hoc test. Mann–Whitney *U* tests were used when comparing DENV vs. iDENV groups from ELISPOT results. *P* values < 0.05 were considered statistically significant.

## 3. Results

### 3.1. Cutaneous DENV Inoculation Induces a Robust Generation of PCs in the DLNs

**Figure 1:**
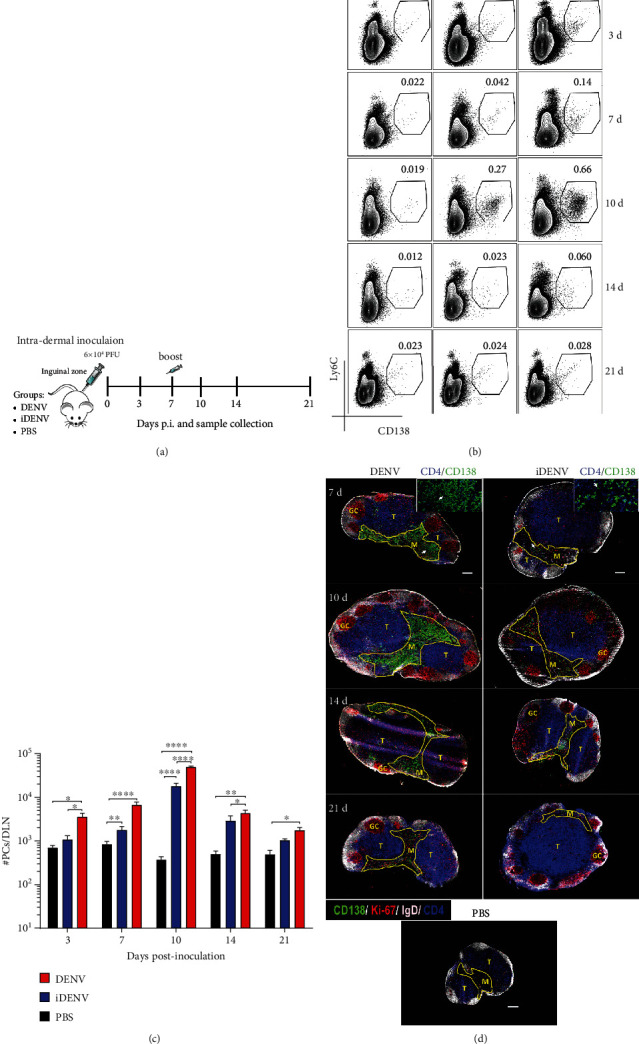
Dynamic of PCs in the draining lymph node (DLN) of immune-competent mice induced by cutaneous DENV inoculation. (a) Mice were inoculated intradermally (i.d.). with 6 × 10^4^ PFU of DENV, UV-inactivated DENV (iDENV), or endotoxin-free PBS at day 0, boosted at day 7, and samples were collected at the indicated days. PCs in the DLN were analyzed by flow cytometry, gating on single/live cells/CD138^+^Ly6C^+^ (Figure S1). (b) Representative contour plots during the kinetic p.i. shows the proportion of PCs with the different conditions. Numbers indicate the proportion of PCs among total single/live cells. (c) Absolute numbers of PCs at days 3, 7, 10, 14, and 21 p.i. (d) DLN cryosections labelled for CD138 (green), CD4 (blue) to identify the T zone (T), IgD for follicular B cells (white), and the proliferation marker Ki-67 (red). GCs are Ki-67^+^ cells inside IgD-negative areas. The medulla (M) is depicted in yellow solid line. Left and right panels show DLNs from DENV- and iDENV-inoculated mice, respectively, whereas the bottom panel shows a representative DLN from noninfected PBS-inoculated mice. A higher magnification of medulla CD138^+^ and CD4^+^ cells is shown for day 7 (white arrow) to illustrate specific staining of CD138^+^ PCs. Scale bars represent 200 *μ*m. Flow cytometry data shown represent the mean ± SEM from at least four independent experiments with two mice per group per time point and histology results from two experiments with four mice per group per time point. Two-way ANOVA with Bonferroni posttest were used for the statistical analysis. ^∗^*P* < 0.05, ^∗∗^*P* < 0.01, ^∗∗∗^*P* < 0.001, and ^∗∗∗∗^*P* < 0.0001.

We have previously shown an increase in the size of DLNs and the induction of B cell responses following cutaneous DENV inoculation in immunocompetent mice [27, 29]. To evaluate the dynamic of PC generation in this model, mice were inoculated i.d. with 6 × 10^4^ PFU of DENV, with a second dose at day 7, and DLNs were collected at days 3, 7, 10, 14, and 21 p.i. of the first dose (Figure 1(a)). We looked for cells concomitantly expressing murine PC markers CD138 and Ly6C [34] in DLNs by flow cytometry (Figure 1(b) and Figure S1). DENV infection led to a significant increase in the percentage and absolute numbers of PCs from day 3 p.i., peaking at day 10 and decreasing progressively from day 14 (Figures 1(b) and 1(c)). UV-inactivated DENV (iDENV), which contains all the viral antigens but is unable to infect and replicate, induced the generation of PCs. However, this response to iDENV was delayed and to a lesser extent compared to active DENV immunization (Figures 1(b) and 1(c)). We assessed *in situ* the distribution of PCs (CD138^+^ cells) at days 7, 10, 14, and 21 p.i. on DLN cryosections (Figure 1(d)). To depict the compartmentalization of DLNs, we stained with anti-CD4 to identify the T cell zone and with anti-IgD and the proliferation marker Ki-67 to identify the B cell follicles and GCs, respectively. At day 7 post-DENV inoculation, CD138^+^ PCs were distributed primarily through the medulla. This pattern was maintained at day 10 but at day 14 p.i., CD138^+^ cells decreased to nearly disappear at day 21 (Figure 1(d), left panel). Following the kinetic with iDENV, CD138^+^ cells were also located mainly in the medullary cords, but again, the extent of this response was lower compared to active DENV (Figure 1(d), right panel). These results show that i.d.-DENV inoculation induces a significant generation of PCs in the DLNs of immune-competent mice.

### 3.2. IgG Class-Switched Cells Dominate the PC Response to Skin-Inoculated DENV

**Figure 2:**
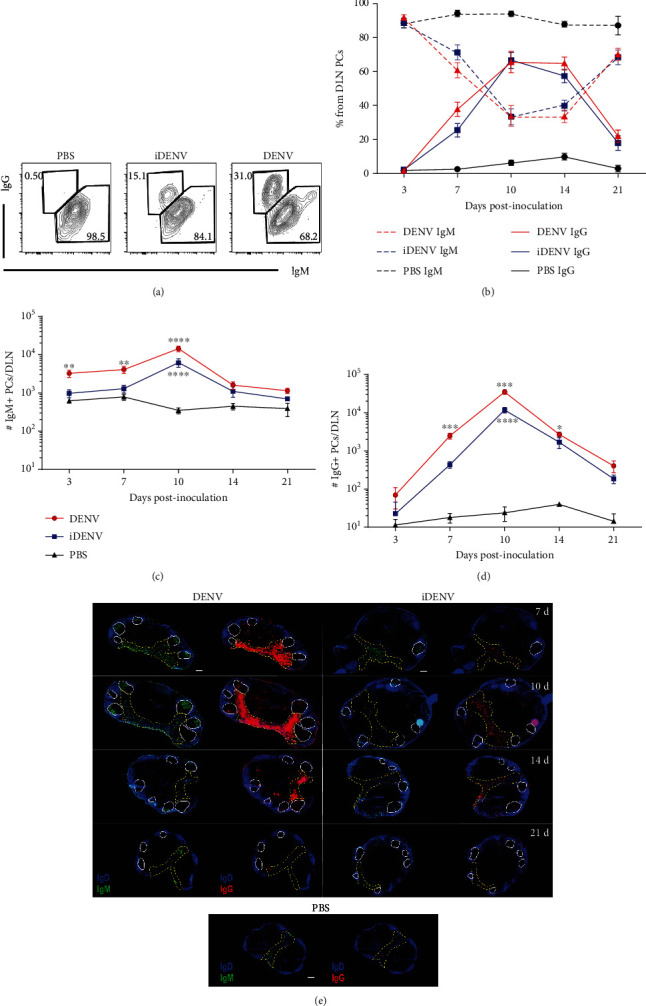
PCs response induced by DENV is largely dominated by IgG^+^ class-switched cells. Class-switched PCs were analyzed by intracellular staining of IgM and IgG by flow cytometry in the DLN of mice at different times postcutaneous inoculation. (a) Representative flow cytometry contour plots of IgM^+^ vs. IgG^+^ PCs (CD138^+^Ly6C^+^) at day 7 post-DENV inoculation. Numbers represent the proportion of IgM^+^ or IgG^+^ cells from PCs. (b) Percentage of IgM^+^ and IgG^+^ PCs during the kinetic. Dotted lines represent IgM^+^ PCs, and solid lines IgG^+^ PCs among the groups. Number of IgM (c) and IgG (d) PCs at days 3, 7, 10, 14, and 21 p.i. (e) DLN cryosections from mice inoculated with DENV (left panel), iDENV (right panel), or PBS (bottom panel) show the distribution of IgM- (green, left) and IgG-expressing cells (red, right). GC zones and the medulla are indicated with white solid lines and yellow dotted lines, respectively. Scale bars represent 200 *μ*m. Data shown represent the mean ± SEM and are representative of four independent experiments with two mice per group per time point and histology results from two experiments with four mice per group per time point. Two-way ANOVA with the Bonferroni post hoc test were used for the statistical analysis. ^∗^*P* < 0.05, ^∗∗^*P* < 0.01, ^∗∗∗^*P* < 0.001, and ^∗∗∗^*P* < 0.0001. Asterisks above the DENV line (solid red line) represent statistical differences against iDENV, and asterisks below the iDENV line (solid blue line) represent statistical differences against the PBS control group.

We then characterized the class-switched PCs post-DENV inoculation analyzing the expression of IgM or IgG antibody classes on CD138^+^Ly6C^+^ PCs by flow cytometry. Class-switched IgG^+^ PCs increased from day 7 p.i. (Figure 2(a)) and peaked at day 10. On the other hand, IgM^+^ PCs decreased from day 7 p.i. in proportion from total PCs (Figure 2(b)). Even though active and inactive DENV induced similar kinetics of the IgM (Figure 2(c)) and IgG class-switched (Figure 2(d)) response, the magnitude was different and significant. Importantly, the IgG^+^ response doubled the one of IgM^+^ (Figures 2(c) and 2(d)). IgG^+^ PCs on PBS control mice were nearly absent as previously reported in peripheral LNs [35]. *In situ* analysis confirmed our findings, showing more of IgG^+^ over IgM^+^ cells and between active and inactive DENV (Figure 2(e)). These results show that PC response to DENV is largely dominated by IgG^+^ class-switched PCs.

### 3.3. Plasma Cell Response to E and prM Viral Proteins Is Similar in DENV Skin-Inoculated Mice

**Figure 3:**
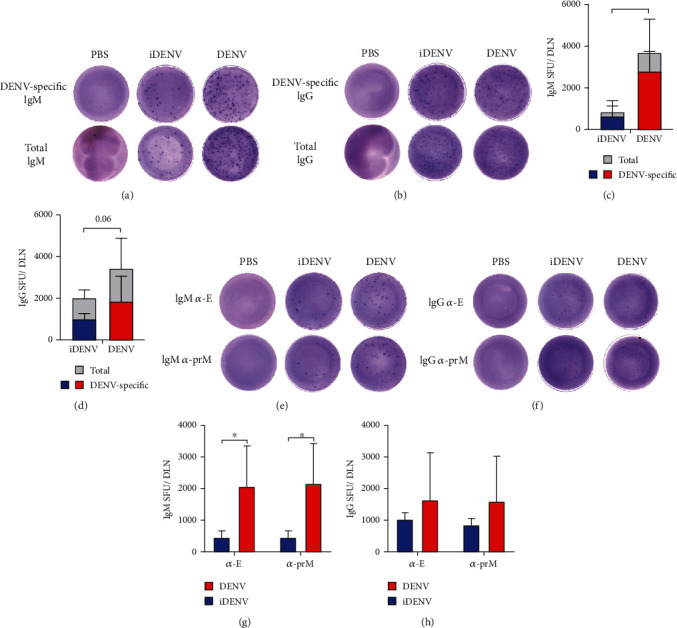
PCs specific to DENV and structural E and prM proteins. ELISPOT assays of DENV-, E-, and prM-IgM and -IgG PCs were performed at day 10 postinoculation. Representative pictures of wells for the detection of total or DENV-specific IgM (a) and IgG SFU (b). Graphs showing the number of total and DENV-specific IgM (c) or IgG SFU (d) per DLN. Representative pictures of wells detecting E- and prM-specific IgM (e) or IgG SFU (f). Graphs showing the number of E- and prM-specific IgM (g) or IgG SFU (h). Data shown represent the mean ± SD of one experiment with 4 mice per group. Data were analyzed with the Mann–Whitney *U* test. ^∗^*P* < 0.05.

Even though we showed an increase of PCs post-DENV inoculation, we wanted to confirm the antigen specificity of the response. To do that, we performed Enzyme-Linked ImmunoSpot assays (ELISPOT) using whole DENV2 as a capture antigen. We confirmed that the increase we showed in PCs at day 10 p.i. was due to the induction of DENV-specific IgM (Figures 3(a) and 3(c)) and IgG (Figures 3(b) and 3(d)) PCs.

The generation of neutralizing and cross-reacting antibodies is important in DENV patients. It has been described that the most potent neutralizing antibodies are directed to certain epitopes on the E protein, while most anti-prM antibodies might be having an infection-enhancing role [9, 14, 36, 37]. To further understand if PCs might be preferentially producing antibodies to E or to prM viral proteins, we evaluated the IgM and IgG PC-specific responses at day 10 p.i.. We found similar numbers of E-specific and prM-specific IgM and IgG SFU in mice inoculated with DENV (Figures 3(e)–3(h)). Consistently with our previous results, iDENV induced a response that is significantly lower than the active virus. Altogether, these results show DENV inoculation induces the generation of virus-specific PCs and importantly, there does not seem to be any difference between the response induced to E and prM viral proteins.

### 3.4. PC Location Correlates with Extrafollicular and GC-Derived Responses, and Most of Them Are Dividing during DENV Infection

**Figure 4:**
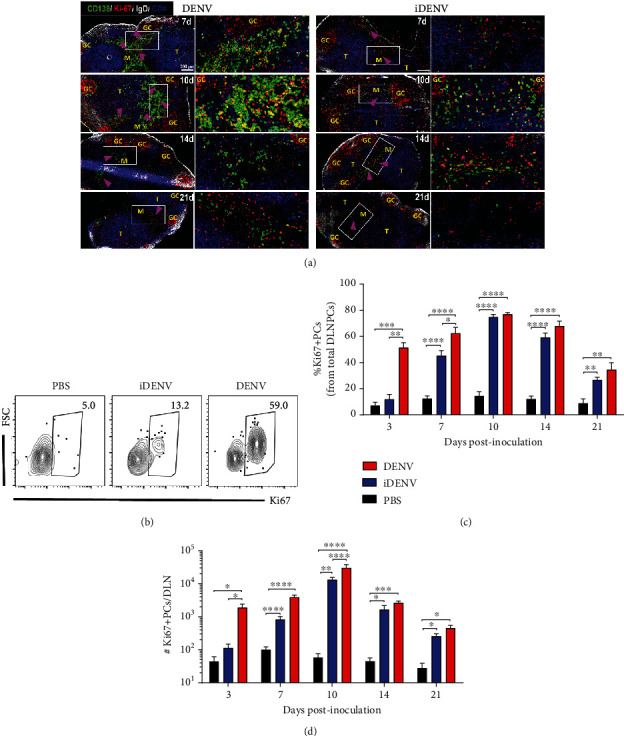
PC distribution and proliferative state during DENV infection. (a) DLN cryosections analyzed at days 7, 10, 14, and 21 postcutaneous inoculation with DENV (left panel) or iDENV (right panel). CD138 (green) antibody-labelled PCs, antibodies for IgD (white), and Ki-67 (red) depict GC zones and CD4 (blue) for T zone (T). Panels on the right of each group correspond to the white squares indicated on the left. PC main location is indicated with purple arrowheads for each time point. Cell suspensions from DLNs were colabelled with antibodies to surface CD138 and Ly6C and intracellularly for Ki-67. (b) Representative flow cytometry contour plots of proliferating Ki-67^+^ PCs at day 3 p.i. Kinetic of the proportion (c) and numbers (d) of Ki-67^+^ PCs in the DLN during the kinetic. Data shown represent the mean ± SEM and are representative of four independent experiments with two mice per group per time point and histology results from two experiments with four mice per group per time point. Two-way ANOVA with the Bonferroni post hoc test were used for the statistical analysis. ^∗^*P* < 0.05, ^∗∗^*P* < 0.01, ^∗∗∗^*P* < 0.001, and ^∗∗∗∗^*P* < 0.0001.

During immune responses, the generation of class-switched extrafollicular plasmablasts (EFPBs) can precede the formation of GCs [21, 22, 38, 39]. In our model, the induction of big and numerous GCs (follicular response) [27, 29] and the appearance of IgG class-switched PCs at later time points post-DENV inoculation (Figure 3) suggest that most of the IgG^+^ PCs are GC-derived. To further analyze their distribution, *in situ* analysis showed clusters of PCs (CD138^+^) in the medullary cords at day 7 p.i. suggesting an extrafollicular response. Furthermore, PCs were also located adjacent to GCs (Ki-67^+^ surrounded by IgD^+^ B cells) in the perifollicular area of the outer T zone called the GC-T interface zone (GTI) (Figure 4(a)) where B cells exit the GC and differentiate [40]. This observation was consistent at day 10. At days 14 and 21, the remaining PCs were distributed mainly in the medulla. In iDENV-inoculated mice, the PCs were also distributed in the medulla (Figure 4(a)). Only at day 10 p.i. some PCs were adjacent to GCs. This analysis showed a substantial frequency of Ki-67^+^ PCs (Figure 4(a)).

We quantified in detail the expression of proliferation marker Ki-67 in PCs by flow cytometry. As early as 3 d post-DENV inoculation, more than 50% of total PCs were Ki-67^+^ (Figure 4(b)). During the kinetic, the percentage and numbers of Ki-67^+^ PCs peaked at day 10 (Figures 4(c) and 4(d)). PCs also expressed Ki-67 post-iDENV inoculation, but the response was delayed and of lower intensity (Figures 4(b)–4(d)). Altogether, these findings show that PCs proliferate in the DLNs during DENV infection, some of them in the GTI border suggesting a GC origin.

### 3.5. Dynamic of Cell Cycle in PCs Induced by Cutaneous DENV Inoculation

**Figure 5:**
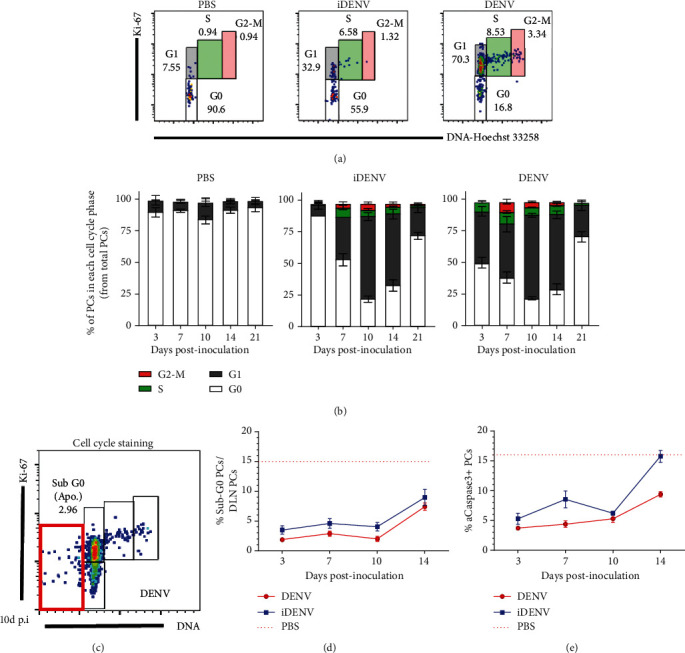
DENV cutaneous infection induces a rapid entrance of PCs to cell cycle and a low apoptosis ratio. For cell cycle analysis by flow cytometry, cell suspensions from DLNs were colabelled with antibodies to surface CD138 and Ly6C and intranuclearly for Ki-67. The DNA was stained with Hoechst 33258. (a) Representative cell cycle gating analysis on PCs from DLN of mice 7 d p.i. with DENV or controls. (b) Percentage of PCs in each cell cycle phase during the kinetic in the DLN of mice postcutaneous inoculation with DENV, iDENV, or endotoxin-free PBS. (c) Representative cell cycle gating strategy on PCs (CD138^+^Ly6C^+^) from a DLN of mice cutaneously inoculated with DENV emphasizing the sub-G0/apoptotic population (red box). (d) Proportion of sub-G0/apoptotic PCs during the kinetic. (e) Proportion of apoptotic PCs expressing the active form of caspase-3 during the kinetic. Red dotted lines in (d) and (e) represent the mean levels in the PBS group. Data shown represent the mean ± SEM and are representative of four independent experiments with two mice per group per time point.

It has been suggested that cell cycle progressively declines once B cells exit the GC and that B cells arrest cell cycle in the G1 phase during the differentiation to PCs [41], but the in-depth analysis of the proliferation capacity of newly generated PCs during ongoing responses *in vivo* has been poorly studied and very limited addressed with infectious antigens. We decided to make use of our model to analyze the expression of the proliferation marker, Ki-67, and the cellular DNA content to identify all cell cycle phases even in small populations and to separate G1 from G0 (quiescent state) [42] (Figure 5(a)). At 3 days post-DENV inoculation, around 40% of total PCs were in the G1 phase and 9% of total PCs were in the S phase (Figure 5(b)). By day 7 p.i., we detected PCs in the G2/M phase. The proportion of PCs in the S and G2/M phases decreased at day 14 p.i. and came back to baseline by day 21. The entry to the G1 and S phase of PCs from iDENV-inoculated mice was delayed until day 7 (Figure 5(b)), and the number of proliferating PCs was significantly smaller compared to DENV during the kinetic (Figure 4(d)).

To assess if the disappearing of PCs from DLNs at day 14 p.i. was due to significant undergoing apoptosis, we analyzed the proportion of PCs in the sub-G0 region of cell cycle (Figure 5(c)) and observed an increase of cells in this phase with both DENV and iDENV (Figure 5(d)). Moreover, we looked for the expression of the active form of caspase-3 (aCaspase3) which is a crucial mediator of apoptosis. In both groups, the proportion of aCaspase3^+^ PCs maintained from day 3 to 10 p.i. and increased at day 14 (Figure 5(e)). Altogether, these results suggest that PCs can divide and expand in their site of generation (DLNs) during DENV infection and a proportion of them undergo apoptosis during the contraction of the response [21, 43, 44].

### 3.6. The Affinity of Anti-DENV IgG Antibodies Increases Progressively Over Time with Neutralizing Capacity

**Figure 6:**
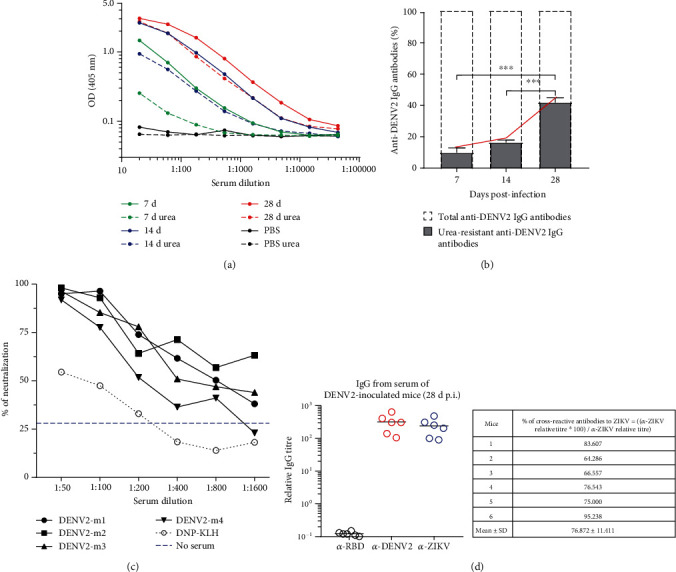
The affinity of IgG antibodies to DENV increases with time after infection, has neutralizing activity, and can be broadly cross-reactive. Sera of mice inoculated with DENV were obtained at days 7, 14, and 28 p.i. and analyzed by ELISA in the presence or absence of urea 7 M. (a) Graph showing the dilution curve of one representative sample per time point. Dashed lines represent the samples with urea 7 M wash. (b) At each time point, the proportion of urea-resistant anti-DENV2 IgG antibodies (high affinity, showed in grey bars) from the total anti-DENV2 IgG antibodies (indicated with dotted bars) is represented. Continuous red line represents the progressive increase in the affinity of the IgG anti-DENV2 antibodies. (c) Percent of neutralization of DENV2 with serum from DENV-inoculated mice (mice 1 to 4, m1-m4) or serum from DNP-KLH-inoculated mice as negative control of neutralization. (d) Relative serum antibody titers for SARS-CoV-2 RBD-, DENV2-, and ZIKV-specific IgG (left panel) and proportion of cross-reactive antibodies to ZIKV from sera of mice 28 d p.i. with DENV2. Data shown represent the mean ± SEM of one experiment with 4 mice per time point (a–c) and two independent experiments with 3 mice per experiment (d) (b, one-way ANOVA test with Bonferroni posttest ^∗∗∗^*P* < 0.001).

Affinity maturation is the key process for the production of high-affinity antibodies and takes place largely on GCs during immune responses [23, 45–47]. To determine if skin-inoculated DENV is inducing high-affinity antibodies, we carried out antigen-specific ELISAs for the detection of anti-DENV IgG antibodies, both in the absence and in the presence of urea 7 M. This assay is used to test antibody affinities and serum avidity in both research and clinical settings [30, 31, 48, 49]. The bound of low-affinity antibodies with the antigen is disrupted under the chaotropic activity of urea while high-affinity antibodies resist this effect and remain bound (Figure S2). We tested the sera of DENV-inoculated mice at days 7, 14, and 28 p.i. to determine the proportion of high-affinity anti-DENV antibodies compared to total anti-DENV IgG antibodies (no urea treatment). At day 7, urea treatment had a pronounced impact as seen with the reduction of the OD in the dilution curve compared to the nonurea counterpart (Figure 6(a)), while this effect was less evident in the serum from mice at days 14 and 28 p.i. The proportion of urea-resistant DENV-specific IgG antibodies increased progressively with time (Figure 6(b)), indicating that high-affinity antibody-producing PCs are generated during DENV infection.

To test if the antibodies generated during infection have neutralizing activity, we tested serial dilutions of sera from mice 28 d p.i. with DENV2 against the homologous virus. We evaluated the percentage of neutralization activity by flow cytometry. The reduction of DENV2 infectivity was about 100% at 1 : 50 dilutions falling to 50% at 1 : 400 dilutions (Figure 6(c)). Representative dot plots of the neutralizing assay are shown in Figure S3. These results were confirmed by plaque reduction neutralization test (Figure S4). Overall, this shows that sera from mice 28 d post-DENV infection have a strong neutralization activity against homotypic infections.

Several studies have shown that DENV infections in humans elicit a wide range of cross-reacting antibodies to heterologous serotypes and also to other mosquito-borne flavivirus with a high degree of sequence and structural homology like ZIKV [9, 33, 50–52]. Thus, to determine if this phenomenon occurs in our model, we carried out antigen-specific ELISAs to detect anti-ZIKV IgG antibodies (Figure S5) and then, we compared the relative titers to anti-DENV IgG antibodies. We found high relative titers of cross-reactive anti-ZIKV IgG antibodies generated in DENV-infected mice. Compared to anti-DENV2 IgG antibodies, this cross-reactivity was about 75% (Figure 6(d)). Nonetheless, this response was flavivirus-specific since no IgG antibodies were detected against nonrelated viral antigens (anti-RBD from SARS-CoV-2).

Altogether, these results show that high-affinity antibodies are produced over time after DENV infection and those antibodies are able to neutralize homologous virus replication. However, these antibodies can also be broadly cross-reactive to related virus such as ZIKV.

## 4. Discussion

Antibodies generated during DENV infection play a key role not only in host protection but also in dengue immunopathology. The preexistence of cross-reactive nonneutralizing antibodies can lead to severe disease during secondary heterotypic infection through enhancement of virus entry on Fc receptor-bearing cells [10, 53, 54]. In addition to this, there is an abundance of proliferating antibody-secreting B cells (plasmablasts), accounting for up to 30% of total peripheral blood mononuclear cells (PBMCs) during acute DENV infection in humans [16, 17, 19]. These two intimately linked characteristics of the immune response to DENV reveal an important protective or pathological role of B cells that is not fully understood.

Very few studies explore PCs (PBs/PCs) in patients with dengue; however, evidence to date demonstrates that cell counts peak in peripheral blood between days 3 and 7 of fever onset and decline suddenly afterwards. Considering that febrile illness presents around 4-10 days after transmission by vector, circulating PCs would appear 1-2 weeks after virus entry through the skin. In order to develop a comprehensive study for the dynamics of PC generation, we used a previously established model of cutaneous DENV infection in immune-competent mice [27].

We evaluated DLNs where PC generation occurs. By flow cytometry, we found a strong response to DENV elicited only in the DLNs that peaked at day 10 p.i. with a contraction at day 14. ELISPOT analysis showed that most of the PC response was DENV-specific, especially of the IgM class. Histological analysis showed that these cells distributed through the medullary cords. This distribution has been shown with model antigens, and it is the site where recently generated PCs receive survival and migratory signals from medullar dendritic cells (DCs), macrophages, and stromal cells [44, 55, 56]. The general location of PCs we found suggests they might be starting the complex process of migration to other niches, possibly the bone marrow [43, 57–59], but this needs further investigation. Nonetheless, an extent of short-lived plasmablasts undergoes apoptosis and is removed by macrophages [60]. In line with this, we also found an increased proportion of CD138^+^ PBs/PCs undergoing apoptosis at day 14 p.i. during the contraction of the response.

It is not clear if PCs during acute DENV infection in humans come from extrafollicular foci reactions or derived from GCs. While IgG^+^ PCs are generally thought to be generated through follicular responses in GCs, two studies in dengue patients hint that the burst of circulating PCs generates extrafollicularly. One suggests the activation of natural already switched IgG B cells and also the activation of polyreactive non-DENV-specific B cells [61]. The second one implies the activation of naïve B cells that switch to IgG and differentiate through Toll-like receptor 7/BCR recognition of DENV, accompanied by later GC–derived PC responses [62]. This latter study is based on the finding of low somatic hypermutation (SHM) rates of antibody genes during acute illness in humans. In line with this, our results show that proliferating CD138^+^ PBs/PCs appears as early as 3 d p.i. which could point out not only to an extrafollicular response but also to polyclonal/unspecific activation triggered by DENV, as it has been shown in humans [61]. This could be further addressed in more detail in this model. At day 7, IgG^+^ PCs are mostly distributed through the medulla, distant from follicles which could indicate also their extrafollicular generation. It has been recently shown by Roco et al. that CSR precedes the formation of GCs [22]. CSR relies on activation of AID which can be induced by TLR7 recognition of viral RNA synergizing with BCR signaling [63]. Our results are compatible with a first wave of IgM and IgG antibody production through early extrafollicular expansion of switched and nonswitched PCs [21, 64]. Further analysis is required to determine the extent of this extrafollicular response compared to PCs from GC origin. The blockade of the interaction between CD40 on B cells and CD40L on T-helper cells with *α*-CD40L antibody treatment would help us to evaluate the GC contribution of PCs in this model [65, 66].

Then, in our study, the response seems to be followed by the generation of GC-derived PCs as hinted by *in situ* analysis where clusters of PCs were observed adjacent to GCs in the GTI zone at days 7 and 10 p.i. (the peak of the response). Although PCs exiting the GC through the GTI border have been described with model antigens such as SRBCs or NP-CGG, to the best of our knowledge, studies with complex infectious microorganisms are absent. Also, the progressive appearance of high-affinity DENV-specific IgGs as shown by avidity ELISA supports a later GC origin. Nevertheless, high-affinity antibody appearance could be also part of extrafollicular PC differentiation [67]. The measure of SHM rates on PCs in our model could also be helpful to characterize the proportion of extrafollicular vs. GC responses. Overall, the importance of virus replication to potentiate both extrafollicular and follicular PC responses is highlighted by the lower extent of PC production seen when DENV is inactivated and unable to replicate.

Of note, consistent with similar IgG titers specific to E and prM viral proteins found in serum [27], we found similar numbers of PCs specific for both viral proteins at the peak of the response. A substantial part of the cross-reactive antibodies generated during a natural DENV infection in humans is directed to both structural proteins. E protein is the main target of neutralizing antibodies; in-depth studies have shown that these antibodies are mainly directed to domain III (EDIII), or quaternary epitopes when dimer or trimer of E proteins are packed on the virion surface, or to the hinge region between EDI and EDII [9, 14, 68, 69]. Nevertheless, antibodies that recognize immunodominant and highly conserved regions like the fusion loop on EDII or to prM protein have poor neutralizing properties but significant infection-enhancing capabilities *in vitro* [9, 14, 37, 70, 71]. In addition, antibodies to prM are produced irrespective of disease severity in humans, in higher quantities during secondary responses, and globally dominates the antibody response against DENV [9, 14, 72]. DENV modulation of the humoral response towards prM antigen has not been studied, but it is possible given the advantage to increase its replication through immature noninfectious virions. The relevance of this has been highlighted by the confirmation of ADE during severe dengue disease in humans [10]. Clearly, to elucidate how DENV and other viruses such as SARS-CoV-2 are inducing a mixture of PCs, some of them producing facilitating antibodies enhancing the probability of subsequent infections [73–75], merits more attention. A limitation to address this in our model would be that immunocompetent mice seem to competently control the viral replication and infection. However, the response and mouse outcome after heterotypic secondary infections have not been tested yet.

Previous studies on IFN-*α*/*β* and IFN-ɣ receptor-deficient (AG129) mice infected with DENV have demonstrated important features of human disease. Also, sequential infection in this model shows a protection to lethal secondary DENV infection through the generation of protective cross-reactive Abs by both MBCs and LLPCs in the spleen and bone marrow [76]. Relevant differences with our work are the use here of immunocompetent mice, a lower viral load inoculated, and the evaluation of DLNs as the site of the response. While mice are neither a natural host nor highly permissive to DENV infection, the strong PC and antibody responses shown in this study raise important questions that could be addressed. One is about the response to heterotypic secondary infections. We showed here the highly neutralizing capacity of the antibodies generated in these mice to homologous DENV *in vitro* but also the generation of cross-reactive antibodies recognizing ZIKV, which share a high homology to DENV. The possible role of these DENV cross-reactive antibodies in ZIKV pathogenesis is still under study in humans [33, 50, 51]. It would be really interesting to test whether mice develop ADE phenomenon to heterotypic infections. We also could evaluate the impact of this secondary infection on mouse physiology (disease signs).

Finally, with this model, we can evaluate the mechanisms behind MBC differentiation (which is relevant for responses to subsequent infections) and analyze the SHM rate and affinity maturation of antibodies, their nonneutralizing vs. protective capabilities, and also the physiological and B cell responses to secondary heterologous infections. In addition, the detailed immune response to prospective vaccines previous to nonhuman primates and human trials could be importantly assessed.

## 5. Conclusions

Altogether, our results show a robust PC response (suggesting both an extrafollicular and a GC origin) to skin-inoculated low-dose DENV in immune-competent mice resembling the findings already described in humans [16–18, 72]. Our observations contribute to the better understanding of B cell responses to DENV. Although mice in this model do not show physiological warning signs of dengue-like disease, the large elicited B cell response can allow the study of the humoral response in an immune unperturbed condition, during the induction and resolution phases of the infection.

## Data Availability

The data used to support the findings of this study are included within the article.

## Supplementary Materials

Supplementary Materials Table S1: antibodies and dyes used for immunofluorescence (IF) and flow cytometry (FCM) staining. Figure S1: gating strategy for the analysis of PC population by flow cytometry. Dot plots show the gating strategy used to identify the PC population in the DLN. CD138^+^Ly6C^+^ double-positive cells were gated from single/live cells. For apoptosis analysis, PCs were gated from total cell population. Numbers indicate the proportion of cells from the gating. Figure S2: affinity of IgG to DNP-KLH increases with time after immunization. Sera of mice inoculated with DNP-KLH were obtained at days 7, 14, and 28 p.i. and analyzed by ELISA in the presence or absence of urea 7 M. (A) Graph showing the dilution curve of one representative sample per time point. Dashed lines represent the samples with 7 M urea wash. (B) At each time point, the proportion of urea-resistant anti-DNP-KLH IgG antibodies (high affinity, show in grey bars) from the total anti-DNP-KLH IgG antibodies (indicated with dotted bars) is represented. Continuous red line represents the progressive increase in the affinity. Data shown represent the mean ± SEM of one experiment with 4 mice per time point. Figure S3: representative dot plots of the neutralization assay by flow cytometry. Twofold serial dilutions of sera from mice 28 d p.i. were mixed with 7.5 × 10^5^ PFU of DENV2 and incubated for 1 h; these complexes were used to infect 2.5 × 10^5^ Vero cells for 24 h, and infection was evaluated by flow cytometry. Sera from DNP-KLH-inoculated mice were used as negative control of neutralization while Vero cells with no serum incubation show the basal level of infection. For analysis, Vero cells were gated from single cells (FSC-H vs. FSC-A). Figure S4: plaque reduction neutralization test. 24-well plates were seeded with Vero cells and when they reached 80-90% confluence; serial dilutions (1 : 20, 1 : 40, and 1 : 80) were made of sera from DENV2-infected mice 28 d p.i. Then, 50 PFU of DENV2 in a volume of 50 *μ*L was added to each serum dilution and incubated for 1 h at 37°C. This serum-virus complex was used to infect the Vero cells in 24-well plates in a final volume of 100 *μ*L for 2 h in triplicate. Sera from DNP-KLH-inoculated mice were used as negative control of neutralization while Vero cells with no serum incubation show the basal level of infection (50 PFU). Figure S5: cross-reactive antibodies to ZIKV from sera of DENV2-inoculated mice at day 28. Serum dilution curves from the experiment are shown. Immunoplates were coated with 2.5 × 10^5^ PFU of either whole DENV2 or ZIKV or 3 *μ*g/mL SARS-CoV-2 RBD overnight at 4°C in a moist chamber. Results shown are from sera of mice 28 d p.i. with DENV2 from two independent experiments with 3 mice (m1-m6) per experiment.
