# Supplementary material for: Robust Plasma Cell Response to Skin-Inoculated Dengue Virus in Mice
Source: J Immunol Res. 2021 Apr 26;2021:5511841. doi: 10.1155/2021/5511841 (PMC8096554; doi:10.1155/2021/5511841)
Supplement: Supplementary Materials — Table S1: antibodies and dyes used for immunofluorescence (IF) and flow cytometry (FCM) staining. Figure S1: gating strategy for the analysis of PC population by flow cytometry. Dot plots show the gating strategy used to identify the PC population in the DLN. CD138+Ly6C+ double-positive cells were gated from single/live cells. For apoptosis analysis, PCs were gated from total cell population. Numbers indicate the proportion of cells from the gating. Figure S2: affinity of IgG to DNP-KLH increases with time after immunization. Sera of mice inoculated with DNP-KLH were obtained at days 7, 14, and 28 p.i. and analyzed by ELISA in the presence or absence of urea 7 M. (A) Graph showing the dilution curve of one representative sample per time point. Dashed lines represent the samples with 7 M urea wash. (B) At each time point, the proportion of urea-resistant anti-DNP-KLH IgG antibodies (high affinity, show in grey bars) from the total anti-DNP-KLH IgG antibodies (indicated with dotted bars) is represented. Continuous red line represents the progressive increase in the affinity. Data shown represent the mean ± SEM of one experiment with 4 mice per time point. Figure S3: representative dot plots of the neutralization assay by flow cytometry. Twofold serial dilutions of sera from mice 28 d p.i. were mixed with 7.5 × 10^5^ PFU of DENV2 and incubated for 1 h; these complexes were used to infect 2.5 × 10^5^ Vero cells for 24 h, and infection was evaluated by flow cytometry. Sera from DNP-KLH-inoculated mice were used as negative control of neutralization while Vero cells with no serum incubation show the basal level of infection. For analysis, Vero cells were gated from single cells (FSC-H vs. FSC-A). Figure S4: plaque reduction neutralization test. 24-well plates were seeded with Vero cells and when they reached 80-90% confluence; serial dilutions (1 : 20, 1 : 40, and 1 : 80) were made of sera from DENV2-infected mice 28 d p.i. Then, 50 PFU of DENV2 in a volume of 50 μL was added [file 5511841.f1.pdf]

Supplementary Material

Table S1. Antibodies and dyes used for immunofluorescence (IF) and flow cytometry (FCM) staining.

Reactivity	Isotype	Clone	Conjugate	Supplier	Use
Active Caspase-3	Rabbit IgG	C92-605	FITC	BD Pharmingen	FCM
CD4	Rat IgG2b, κ	RM4-4	PE	BioLegend	IF
CD4	Rat IgG2b, κ	GK1.5	APC	eBioscience	IF
CD138	Goat IgG	Polyclonal	Purified	R&D Systems	IF
CD138	Rat IgG2a, κ	281-2	PE	BD Pharmingen	FCM
Goat IgG	Donkey IgG	Polyclonal	Alexa Fluor 488	Jackson Immunoresearch	IF
Goat IgG	Donkey IgG	Polyclonal	Alexa Fluor 555	Invitrogen	IF
IgD	Rat IgG2a, κ	11-26c.2a	Brilliant Violet 421	BioLegend	IF
IgG	Goat IgG F(ab')2	Polyclonal	APC	eBioscience	IF/FCM
IgM	Goat IgG	Polyclonal	FITC	Southern Biotech	IF/FCM
Ki-67	Rabbit IgG	Polyclonal	Purified	Abcam	IF
Ki-67	Mouse IgG1, κ	B56	Alexa Fluor 488	BD Pharmingen	FCM
Ly-6G/Ly-6C	Rat IgG2b, κ	RB6-8C5	PerCP	BioLegend	FCM
Rabbit IgG	Donkey IgG	Polyclonal	Cy3	Jackson Immunoresearch	IF
Streptavidin			PE-Texas Red	BD Pharmingen	FCM
Hoechst 33258				Polysciences	FCM
Fixable Viability Dye			eFluor 450	eBioscience	FCM

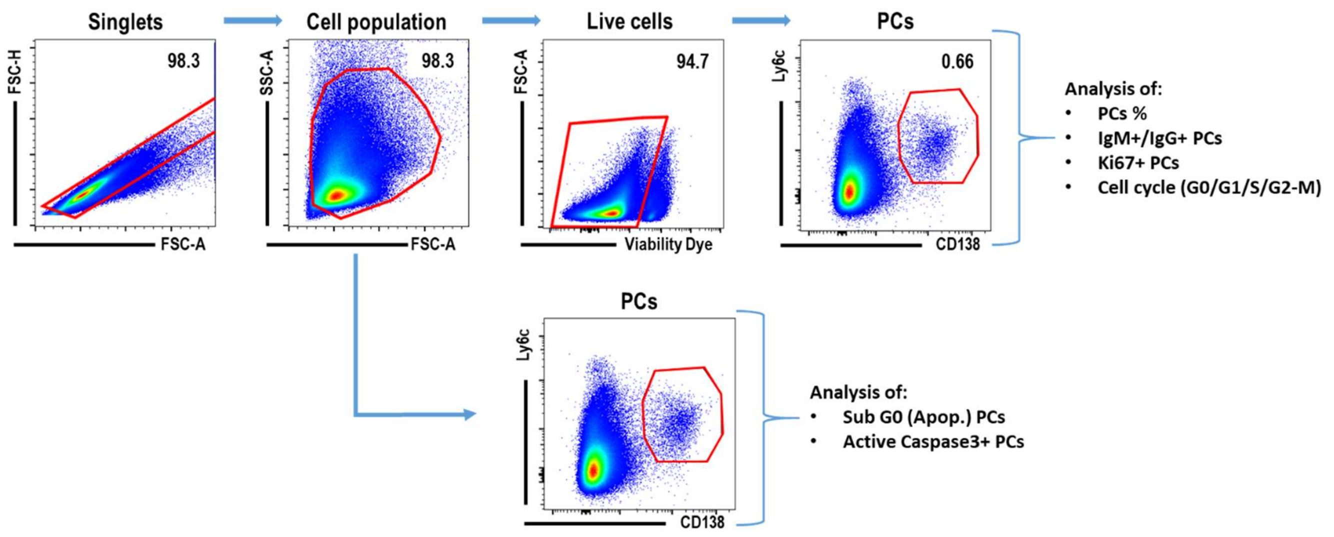

Figure S1. Gating strategy for the analysis of PC population by flow cytometry. Dot plots show the gating strategy used to identify the PCs population in the DLN. CD138^{+} Ly6C^{+} double positive cells were gated from single/live cells. For apoptosis analysis, PCs were gated from total cell population. Numbers indicate the proportion of cells from the gating.

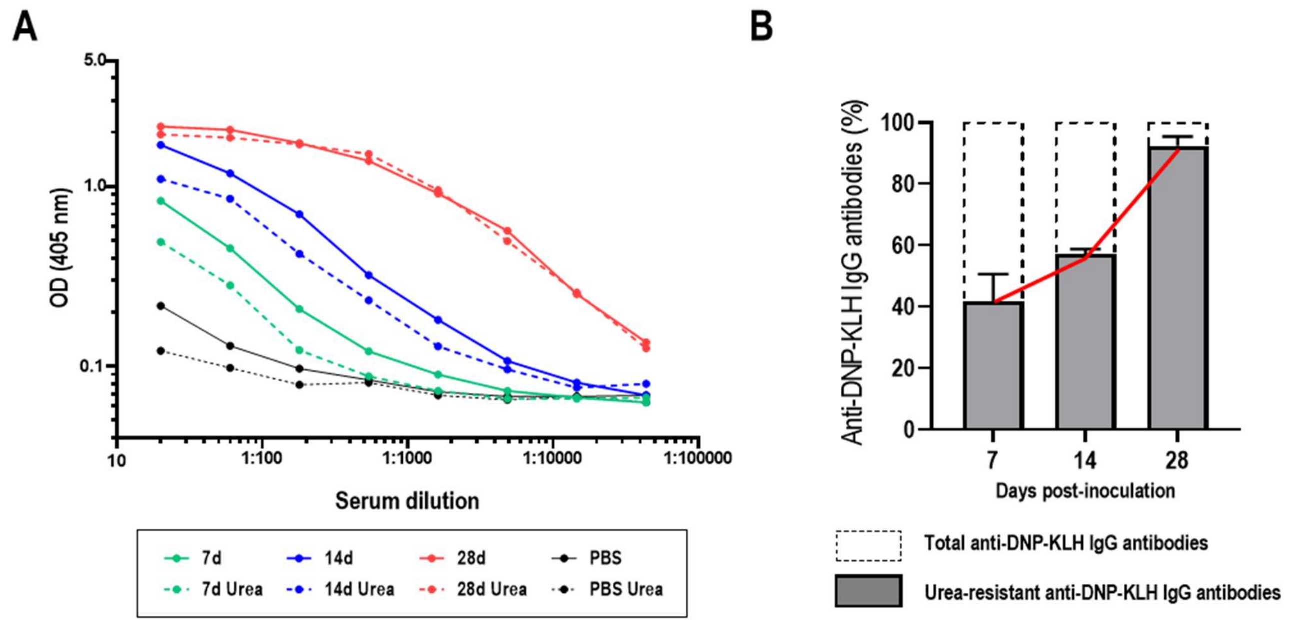

Figure S2. Affinity of IgG to DNP-KLH increases with time after immunization. Sera of mice inoculated with DNP-KLH were obtained at days 7, 14 and 28 p.i. and analyzed by ELISA in the presence or absence of urea 7M. **(A)** Graph showing the dilution curve of one representative sample per time point. Dashed lines represent the samples with 7M urea wash. **(B)** At each time point, the proportion of urea-resistant anti-DNP-KLH IgG antibodies (high affinity, show in grey bars) from the total anti-DNP-KLH IgG antibodies (indicated with dotted bars) are represented. Continuous red line represents the progressive increase in the affinity. Data shown represent the mean ± SEM of one experiment with 4 mice per time point.

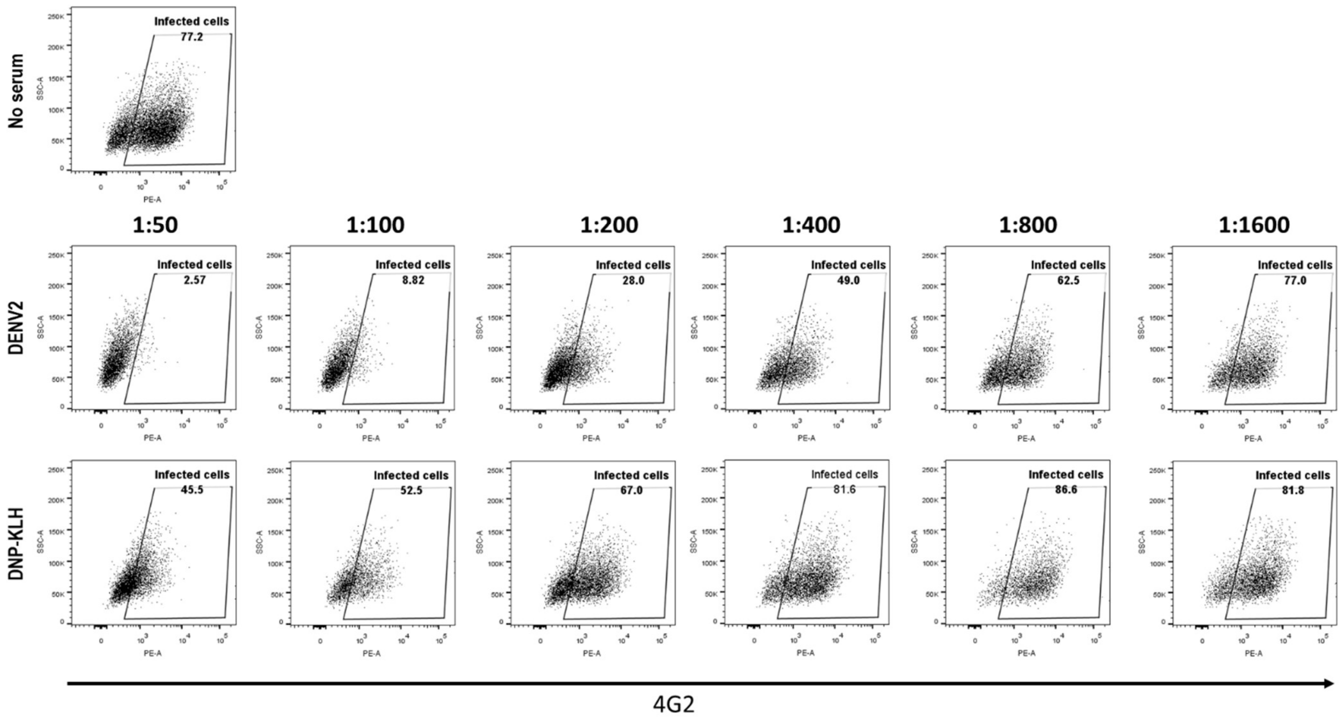

Figure S3. Representative Dot plots of the neutralization assay by flow cytometry. Two-fold serial dilutions of sera from mice 28d p.i. were mixed with 7.5 x 10^5 PFU of DENV2 and incubated for 1h; these complexes were used to infect 2.5 x 10^5 Vero cells for 24h, and infection was evaluated by flow cytometry. Sera from DNP-KLH-inoculated mice were used as negative control of neutralization while Vero cells with no serum incubation shows the basal level of infection. For analysis, Vero cells were gated from single cells (FSC-H vs FSC-A).

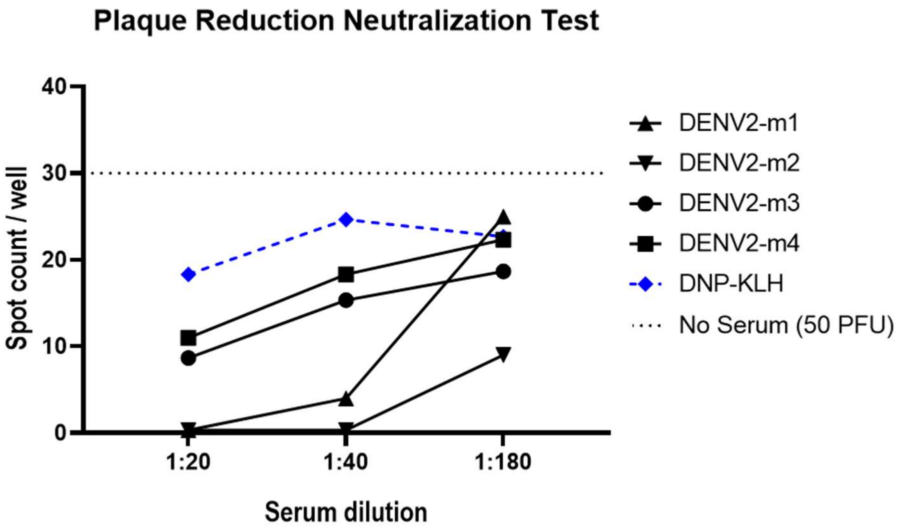

Figure S4. Plaque Reduction Neutralization Test. 24 well plates were seeded with Vero cells, and when they reached 80-90% confluence; serial dilutions (1:20, 1:40 and 1:80) were made of sera from DENV-2 infected mice 28d p.i.. Then, 50 PFU of DENV2 in a volume of 50ul was added to each serum dilution and incubated for 1h at 37°C. This serum-virus complex was used to infect the Vero cells in 24 well plates in a final volume of 100ul for 2h in triplicates. Sera from DNP-KLH-inoculated mice were used as negative control of neutralization while Vero cells with no serum incubation shows the basal level of infection (50 PFU).

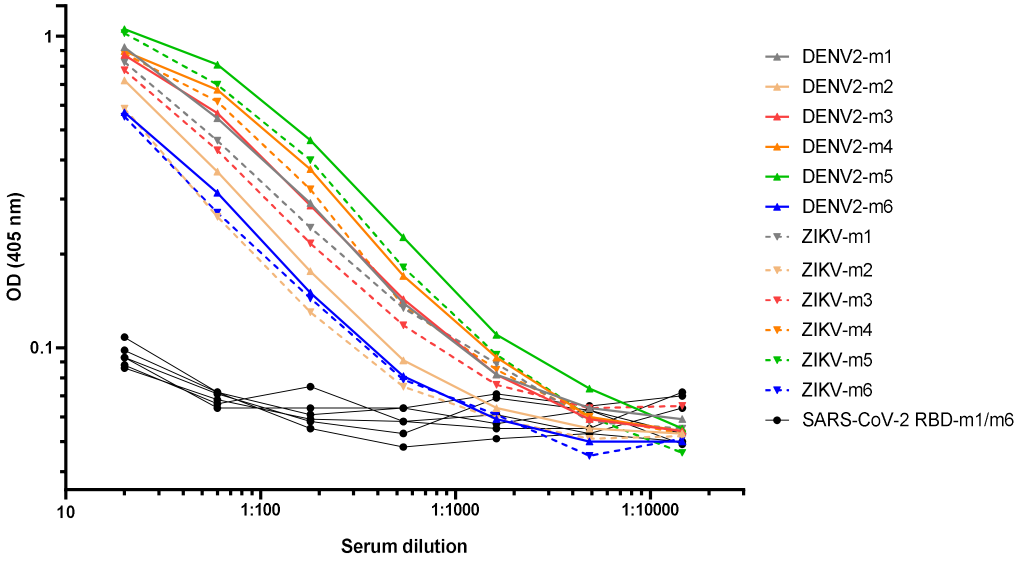

Figure S5. Cross-reactive antibodies to ZIKV from sera of DENV2 inoculated mice at day 28. Sera dilutions curves from the experiment are shown. Immunoplates were coated with 2.5 x 10^5 PFU of either whole DENV2 or ZIKV or 3 µg/mL SARS-CoV-2 RBD overnight at 4°C in a moist chamber. Results shown are from sera of mice 28d p.i. with DENV2 from two independent experiments with 3 mice (m1-m6) per experiment.
